# Dynamic modulation of frontal theta power predicts cognitive ability in infancy

**DOI:** 10.1016/j.dcn.2020.100818

**Published:** 2020-07-08

**Authors:** Eleanor K. Braithwaite, Emily J.H. Jones, Mark H. Johnson, Karla Holmboe

**Affiliations:** a Centre for Brain and Cognitive Development, Birkbeck, University of London, United Kingdom; b Department of Psychology, University of Cambridge, United Kingdom; c Department of Experimental Psychology, University of Oxford, United Kingdom

**Keywords:** Frontal theta oscillations, Cognitive development, Non-verbal ability, Executive function, EEG, Neural correlate

## Abstract

Cognitive ability is a key factor that contributes to individual differences in life trajectories. Identifying early neural indicators of later cognitive ability may enable us to better elucidate the mechanisms that shape individual differences, eventually aiding identification of infants with an elevated likelihood of less optimal outcomes. A previous study associated a measure of neural activity (theta EEG) recorded at 12-months with non-verbal cognitive ability at ages two, three and seven in individuals with older siblings with autism (Jones et al., 2020). In a pre-registered study (https://osf.io/v5xrw/), we replicate and extend this finding in a younger, low-risk infant sample. EEG was recorded during presentation of a non-social video to a cohort of 6-month-old infants and behavioural data was collected at 6- and 9-months-old. Initial analyses replicated the finding that frontal theta power increases over the course of video viewing, extending this to 6-month-olds. Further, individual differences in the magnitude of this change significantly predicted non-verbal cognitive ability measured at 9-months, but not early executive function. Theta change at 6-months-old may therefore be an early indicator of later cognitive ability. This could have important implications for identification of, and interventions for, children at risk of poor cognitive outcomes.

## Highlights

• This longitudinal infant study found that frontal theta power increased over the course of video viewing in 6-month-olds.
• Individual differences in frontal theta power change at 6-months-old significantly predicted non-verbal cognitive ability.
• Individual differences in frontal theta power change at 6 months was not associated with executive function at 9 months.
• Frontal theta change at 6 months may be an early indicator of later cognitive abilities.

## 1 Introduction

Identifying the mechanisms that underpin individual differences in cognitive functioning in infancy could lead to substantial theoretical insights into the developmental origins of core cognitive skills, and could improve our ability to rapidly identify children at heightened risk for poor cognitive development. When searching for early predictors of later cognitive functioning, behavioural measures are often confounded by factors such as language, motor and attention capacities and can be hard to apply across cultures (Lloyd-Fox et al., 2014b). Neural measures may be a more fruitful avenue for investigation that also allow greater mechanistic insight. Electroencephalography (EEG) is a method which measures oscillations in neural processing associated with information consolidation in learning and memory (Assenza and Di Lazzaro, 2015). It is non-invasive and therefore suitable for use with infants and young children. Electrical oscillations gathered using EEG can be segmented and separated into frequencies, providing a specific signal within each broad neural region which may be linked to cognitive functions.

In this paper, we focus on an analysis of power within the theta band frequency recorded over frontal channels, since increases in theta power (particularly low-range theta and over frontal brain areas) have been strongly associated with learning and memory functions across species (Hsieh and Ranganath, 2014). Hippocampal theta has also been implicated in information coding and memory function across species and is thought to drive oscillations in frontal and temporal brain regions (see, Lega et al., 2012, for review). Early animal work indicated that hippocampal theta may be particularly important for learning and memory development. Long-term potentiation, considered a model of learning, is dependent on the particular phase of hippocampal theta activity, with theta oscillation peaks associated with enhanced synaptic plasticity and troughs with decreased synaptic plasticity (Hölscher et al., 1997). An increase in theta power across frontal regions has been linked to memory maintenance (Jensen and Tesche, 2002), memory encoding (Long et al., 2014) and processing of unexpected auditory input (Hsiao et al., 2009) in human adults.

Similar results demonstrating an increase in theta power across frontal regions during activities requiring considerable cognitive processing have been found in young children (i.e., during toy exploration and communication with an adult), indicating that theta function may be somewhat analogous across animals, human adults and infants (Orekhova et al., 2006). In line with adult findings relating to episodic memory (see, Sederberg et al., 2003, for discussion), Begus, Southgate, and Gliga (2015) found that increases in frontal theta oscillations during object exploration correlated with subsequent recognition of that object in infants aged 11 months. Notably, this effect was specific to frontal regions and the theta frequency, and was not mediated by extent of visual or physical exploration, indicating that behavioural attention measures may not capture this learning as well as neural measures. Frontal theta oscillations have also been associated with other situations likely to involve infant learning, including during the anticipation stage of a peek-a-boo game (Orekhova et al., 1999), novel toy exploration, attention to social stimulation (Orekhova et al., 2006), and when expectations are violated (Berger et al., 2006; Köster et al., 2019 (although here the effect pertained to visually entrained posterior theta oscillations)).

In many previous studies, participants completed experimental tasks with different conditions and theta power was compared between these conditions. However, within what are sometimes thought of as ‘baseline’ recordings, changes in frontal theta power in infants have also been found to be informative (see Camacho et al., 2020, for a discussion of the importance of considering the resting baseline in young children). For example, Stroganova and Orekhova (2007) found increases in frontal theta power in a group of 7- to 11-month-old infants whilst they watched an experimenter blow soap bubbles. Infants watched for one minute and analyses comparing 20-second segments of theta power sampled during the first 40 s and second 40 s of bubble blowing revealed that frontal theta power in the latter period was higher than in the former. This suggests that learning and information consolidation may increase as infants continue to watch engaging stimuli and supports the potential occurrence of interesting and notable processes within ‘baseline’ periods. Similarly, a recent study investigating theta oscillations in pre-schoolers indicated that theta power over fronto-central regions increased across multiple rest periods (a fixation cross) interspersed between cognitive tasks (Meyer et al., 2019), supporting an association between theta modulation and engagement of cognitive processes. However, it is important to note that for infants the distinction between task-present and task-absent (‘baseline’) epochs of an experiment is limited—without verbal instruction, the whole session is a series of experiences during which their brain activity is measured. Changes in frontal theta power during periods of passive observation in infants may thus offer insight into individual differences in infants’ early emotional and cognitive processing of stimuli.

The cognitive mechanisms associated with theta power (as with all oscillations) remain debated (Colgin, 2013; Herrmann et al., 2016). In part, this reflects the broader challenge of accurately integrating neural and cognitive models of the brain, and perhaps indicates that our conceptualisations at the two levels are mismatched. However, several lines of research do indicate that the theta rhythm is related in important ways to a range of cognitive processes, such as learning, memory and attention. Miller (1991), for example, proposed that theta plays a role in the interplay between cortical and limbic structures and may reflect a somewhat general process related to the encoding and retrieval of information. Klimesch (1999) also purports a strong relation between theta power and information encoding, based upon evidence supporting a link between synchronous activity of hippocampal theta and long-term potentiation. Theta rhythm is not only found during memory-related tasks however, it is also present during other cognitive states and behaviours, such as expectation of painful stimuli in adults (Kornhuber et al., 1990) and sucking behaviour in infants (Futagi et al., 1998). Increases in theta may thus not be specific to memory functioning but may also be related to other functions. As discussed above, some work indicates a relation between frontal theta increases and attention, especially in infants (Stroganova and Orekhova, 2007). One unifying possibility is that theta represents a general mechanism of information processing. That is, increased theta may improve the processing of a particular visual (or other) stimulus and protect against the interference of other signals (Vinogradova et al., 1998). This increased processing of information – be that of a visual stimulus, a representation in memory, or some other cognitive phenomenon – may lead to enhanced learning and functioning across a number of cognitive domains. In sum, although its precise functions remain debated, evidence indicates a role for theta oscillations in underpinning learning and memory.

A complement to experimental attempts to dissect the precise cognitive ‘meaning’ of theta oscillations is to examine whether they have predictive validity to later cognitive outcomes. Whilst broad changes in theta power have been associated with information consolidation, few studies have focussed on whether individual differences in theta change or modulation associate with later developmental outcomes. A recent study in a sample consisting primarily of infants with older siblings with autism (Jones et al., 2020) found that an increase in frontal theta power over the course of video-viewing at 12 months significantly predicted cognitive outcome in the same children at ages 2, 3 and 7 years. However, at present it is unknown whether the same predictive effect of frontal theta change extends to a purely low-risk sample and whether it can be observed even earlier in infancy.

The present pre-registered study sought to address these questions. Furthermore, there is a large literature linking fronto-medial theta in adults to cognitive control (Cavanagh and Frank, 2014; Clayton et al., 2015), as well as direct evidence linking theta band activity to executive functioning (EF) later in childhood (Perone et al., 2018). Meyer et al. (2019) presented evidence that frontal theta is involved in top-down control during task engagement as well as across multiple stimulus presentations in 4-year-olds, and frontal theta activity has been associated with error monitoring, a function linked to cognitive control, in toddlers (Conejero et al., 2016). Finally, Stroganova and Orekhova (2007) speculated that the predominantly frontal theta activation in 7- to 11-month-old infants during attention-eliciting episodes could indicate the engagement of frontally-mediated executive processes. One possibility is that the association between frontal theta modulation and cognitive skills is mediated through better EF, and so we additionally asked whether frontal theta change was related to early indicators of EF.

### 1.1 The present study

The present study utilised EEG and behavioural data from a large sample of typically developing infants tested longitudinally (Holmboe et al., 2010, 2018a). We examined individual differences in the increase in theta power observed whilst infants viewed a dynamic video, as this type of viewing has been previously linked to enhanced neural activation during internal control of attention, learning and memory formation (Strogonova & Orekhova, 2007). Previous work has considered frontal theta power change whilst infants watch an experimenter blowing soap bubbles, however, the use of a video of bubbles, vehicles and other similar stimuli during EEG recording enables greater experimental control and consistency across infants.

We had three aims in this study. First, we examined the robustness of a refined operationalisation of individual differences in frontal theta change (the degree of change in frontal theta power over the course of video viewing). We included two indices of frontal theta change; a measure of change from the first 30 s to the second 30 s of video viewing, and a continuous measure of change throughout the video. The former index is in line with previous work (Stroganova and Orekhova, 2007; Jones et al., 2020), whilst the latter allowed us to reduce the relatively high drop-out rate reported by Jones et al. (2020) and is theoretically motivated by the potential for individual variation in the time-frame of dynamic changes in EEG. That is, theta power for some individuals may significantly increase after 20 s of video viewing, whilst for others this may occur after 50 s, therefore, the second index allows for additional differences in theta power change to be detected across participants.

Second, we tested whether there was a positive predictive relationship between frontal theta power change at 6 months and non-verbal ability, as assessed by the Mullen Scales of Early Learning, American Guidance Service edition (MSEL-AGS; Mullen, 1995) at 9 months. In accordance with the pre-registration (Holmboe et al., 2018b), the included measures from the MSEL-AGS were the same as those found to be associated with frontal theta power change in a population at high risk for Autism Spectrum Disorder by Jones et al. (2020) and did not include other MSEL-AGS scales; only non-verbal scales were included to limit the number of statistical analyses to the hypothesised effects. Verbal scales are likely less relevant in this sample, since communication skills are only just emerging at 9 months old. Further, Jones et al. (2019) found that expressive and receptive language scores at 5 and 10 months of age were subject to more site effects than the other MSEL scales in a multi-site study, indicating that these scales may be somewhat less robust.

Third, we tested whether there were positive associations between individual differences in frontal theta power change and indicators of executive functioning (EF) at both 6 and 9 months. To assess early EF, we used the A-not-B task (requiring both inhibition and working memory; Diamond, 1985; Bell and Fox, 1992) and the Freeze-Frame task (a saccade-based measure of inhibition; Holmboe et al., 2008).

## 2 Method

Our analyses of the Holmboe (2017, https://thesiscommons.org/qe9ck/, thesis originally submitted in 2008) dataset were pre-registered on the Open Science Framework website. Extensive details on our analysis plan, indices and exclusions can be found in the pre-registration: https://osf.io/v5xrw/. Importantly the EEG analyses were carried out separately by a different team (E. K. B. and E. J. H. J.) from the behavioural testing and data analyses (K.H.) and the two datasets were only combined after the pre-registration had been submitted.

### 2.1 Participants

Parents and infants were recruited from a lab database when infants were 4 months old. Parental informed consent was obtained before participation in the study. The whole cohort included 104 (51 male, 53 female) four-month-old infants from the Greater London area, with 94 retained at the 9-month visit. Most visits occurred within one week of infants reaching their 6-/9-month birthday. Of this group, 47 infants provided sufficient artefact-free EEG data at 6 months and 45 (28 female) of these also had sufficient behavioural data to be included in the analyses. Over three quarters of the sample was of White ethnic background (80 %), 15.6 % were of Mixed ethnic background, 2.2 % were of Asian ethnic background and 2.2 % were of Black ethnic background. Table 1 shows descriptive statistics for this sample. Behavioural data from the 6-month and 9-month visits have been reported previously (Holmboe et al., 2018a), but this is the first time the EEG data has been analysed and reported. The original study and this analysis received ethical approval from the Department of Psychological Sciences ethics committee at Birkbeck (ref. no. 2248).

**Table 1:** Demographic data for the full sample included in this study.

	*n*	Mean	*SD*	Minimum	Maximum
Infant age in days (6 m)	45	182.02	5.43	174	196
Infant age in days (9 m)	42	274.6	6.49	266	295
Mother’s age in years	42	34.86	5.71	21	47
Mother’s years of education	41	17.8	2.74	13	26
Father’s age in years	41	35.56	6.05	23	52
Father’s years of education	39	17.0	2.18	11	21

### 2.2 Measures

#### 2.2.1 EEG

##### 2.2.1.1 Apparatus and stimuli

EEG was recorded in a shielded room using 64-channel sensor nets from Electrical Geodesics Inc. (EGI), referenced to the vertex, digitised at 250 Hz, and band-pass filtered at 0.1–100 Hz.

##### 2.2.1.2 Procedure

Infants were presented with non-social videos (e.g., abstract moving shapes, leaves falling; SM 1.1.1 & 1.1.2) combined with a simple tune, during which EEG was recorded. The total length of the videos was 2 min and 14 s, however, since this video was used as a precursor to another task, video session time varied, ranging between 105 and 165 s (*M* = 121.11, *SD* = 10.65). Video session time as reported here did not take into account whether the infant watched or not, however, separate measures of on-screen looking time were calculated and analysed; these analyses are presented in the Supplementary Materials (SM 2.4 & 2.5). Due to technical difficulties, the stimulus video was stopped and restarted for two participants, meaning that total video session time was longer than the length of the video for those participants. Videos were coded offline using *Datavyu* to ensure that infants were attending to the screen for the majority of the time. Over the course of video presentation, videos were coded according to whether the infant’s eye-gaze was upon the screen or elsewhere. Percentages were then calculated according to how many milliseconds were spent with eye-gaze on the screen versus elsewhere during video viewing time. All participants included in the analysis looked at the screen for at least 60 % of the time, in line with similar neuroimaging work by Lloyd-Fox and colleagues (Lloyd‐Fox et al., 2009; 2014a).

##### 2.2.1.3 EEG analysis

EEG data was segmented into 1-second segments, artefacts were removed (NetStation) and a Fast Fourier Transform (Matlab) was used to extract power in the 3−6 Hz band across fronto-central electrode sites (SM 1.1.3, Figure S1). Though there are some inconsistencies in what frequency range constitutes infant theta, 3−6 Hz is the most commonly used range (Saby and Marshall, 2012). In accordance with the pre-registration, two indices of change in theta power were then calculated.

For Index A: The first 1 minute of data from the first clean segment of data was used; this was split into two 30-second halves for comparison. Participants were only included if they provided at least five artefact-free segments per first and second 30 s of this period. Inclusion criteria for both indices are in line with previous work (Michel et al., 2015; Elsabbagh et al., 2009; Southgate et al., 2008). Power values were averaged across artefact-free segments and electrodes within a fronto-central topographical group (see Figure S1) and within each of the first and second 30 s of the video. Thirty seconds was chosen for this index in line with Jones et al. (2020), who compared the first and second halves of a one-minute long video. Natural logs were calculated to reduce skew. Logged power values were then averaged across the theta (3–6 Hz) frequency range. Theta power change of Index A was calculated as the difference between average power in the first 30 s of calm video viewing (operationalised from the first clean segment of EEG data) from theta power in the next 30 s. Index B was calculated because Index A resulted in a relatively high drop-out rate in previous work and to account for potential individual differences in the time-frame of frontal theta change.

For Index B: Participants were only included if they provided at least 10 artefact-free segments over the course of the whole video (from the first clean segment). Theta power for each artefact-free segment was calculated by averaging across electrodes within the topographical group as in Index A. These were correlated with segment number using Pearson correlation to establish the rate of theta power change over the course of the video for each infant. Segments were numbered from the first clean segment of data. We first established that Index B was sensitive to the positive association between theta increase and ‘time’ as observed in previous work (Jones et al., 2020) and produced data for a higher number of infants; that index was then used in all further analyses. ‘Time’ here refers to how long the infant had been presented with the video for, from the first clean segment of data; as described above, only infants who were looking at the screen at least 60 % of the time were included in the analysis. For full details on the EEG analysis, see SM 1.1.

### 2.3 Measures of executive functioning

#### 2.3.1 The Freeze-Frame task

##### 2.3.1.1 Procedure

The Freeze-Frame task (Holmboe et al., 2008, 2018a) was administered at both 6 and 9 months, with a slightly modified, simpler version used at the younger age point. Briefly, infants watched dynamic cartoon animations in the centre of the screen. On every trial, a white distractor square was presented peripherally on the left or right side of the screen. The duration of the distractor was individually calibrated in 40-ms steps, starting at 200 ms, until the infant looked to the distractor on two consecutive trials, at which point the distractor duration was fixed. The main difference between the 6-month and 9-month version of the task was that the 9-month version involved a mix of interesting (engaging central stimulus) and boring (repetitive central stimulus) trials, whereas the 6-month version only included interesting trials (for full details, see Holmboe et al., 2018a, which can be freely accessed at https://psyarxiv.com/psb8f/).

##### 2.3.1.2 Individual performance measure

The Freeze-Frame Inhibitory Control index was the proportion of looks to the distractor across all trials at 6 months and in interesting trials only at 9 months (the trial type that most closely matched the 6-month version).

#### 2.3.2 The A-not-B task

##### 2.3.2.1 Procedure

The A-not-B task was administered at 9 months. The version of the A-not-B task used in this study involved an adaptive testing procedure where the hiding location and delay between hiding and search were adjusted depending on the infant’s performance (for full details, see Holmboe et al., 2018a). Briefly, on each trial the experimenter hid a toy in one of two wells in a table in front of the infant and covered the wells with cardboard squares; infants were then distracted during an imposed variable-length delay. The initial delay was set to 2 s. The experimenter hid the toy in the same location (starting at Location A, counterbalanced across participants) until the infant had successfully found the toy at that location on two consecutive trials. At this point, the toy was hidden in the other well (change trial), and then was repeatedly hidden in that well until the infant had completed another two successful trials consecutively. If two consecutive trials ended in failure, the delay period was decreased by 2 s. If two consecutive change trials ended in success, the delay period was increased by 1 s. Infants were encouraged to complete 40 A-not-B trials. A second experimenter, out of view from the infant, used a computer to score the infant’s responses online and to indicate to the first experimenter what delay duration should be used on each trial and when the delay period was over (a delay count-down was shown to the first experimenter on a computer screen behind the infant to keep timings precise).

##### 2.3.2.2 Individual performance measure

The maximum delay that infants could sustain on change trials was used in the analyses. This was the same measure as used by Holmboe et al. (2018a).

#### 2.3.3 Mullen Scales of Early Learning at 9 months

The Mullen Scales of Early Learning, AGS Edition (MSEL-AGS) is an assessment of children’s motor and cognitive development that can be used from birth to five years of age (Mullen, 1995). It consists of five scales: Gross Motor, Visual Reception, Fine Motor, Receptive Language, and Expressive Language. A trained experimenter administered the assessment to children individually, following the Freeze-Frame and A-not-B tasks. The assessment was scored during the session and was also recorded on video. The item scores were checked and corrected (if needed) by a second trained scorer offline before scale scores were calculated.

##### 2.3.3.1 Individual performance measures

Standardised scores for the five scales were calculated according to the manual (Mullen, 1995). Only a measure of non-verbal cognition was used in the confirmatory analyses; this was calculated by averaging the standardised scores for the Visual Reception and Fine Motor scales (Bishop et al., 2011). Exploratory analyses were carried out on the Visual Reception and Fine Motor scales separately.

### 2.4 Correlation analyses

Correlations were used to assess bivariate associations between frontal theta power change and Freeze-Frame Inhibitory Control score at 6 and 9 months, A-not-B score at 9 months and MSEL non-verbal score at 9 months. Positive associations were predicted in all analyses (i.e., higher theta change would be associated with better EF and cognitive performance). In line with the pre-registration (http://osf.io/v5xrw), a one-tailed p-value of less than 0.05 was used to infer statistical signiﬁcance for these confirmatory analyses. Two follow-up exploratory correlation analyses were also carried out. A Bonferroni correction for multiple comparisons was applied for the exploratory analyses such that a two-tailed p-value of less than 0.025 was taken to be suggestive of a significant effect.

## 3 Results

### 3.1 Identifying the best index of change in frontal theta power

To identify the best index of change in frontal theta power, we followed the steps we set out in the pre-registration. Index A (n = 33, 35.5 % of the full sample who came for the visit at 6-months, 34.0 % of the full sample from whom some EEG was gathered) compared average theta power in the first and second 30 s of video viewing (for details, see EEG section under Method), with theta power change calculated as the difference between the two. A one-sample *t*-test showed that theta power change for each participant was not significantly different from zero *t*(32) = 1.01, *p* = 0.321, 95 % CI [−0.03, 0.10], *M* = 0.03, SD = 0.19.

**Fig. 1:**
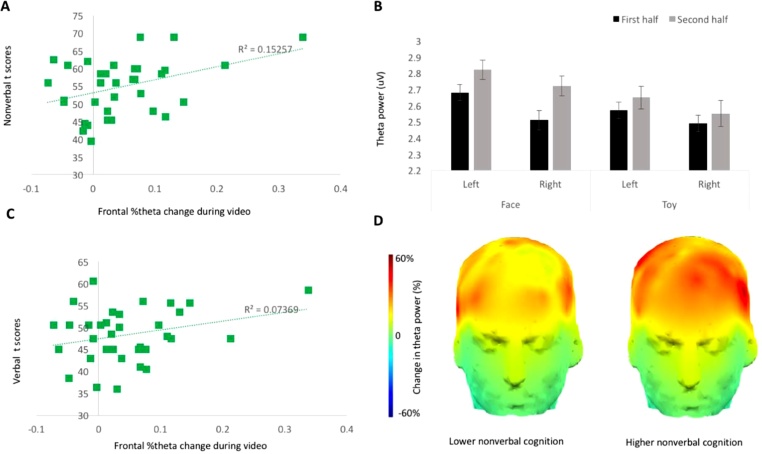
Data from the collapsed sample, generated by averaging frontal theta power data from each infant who provided a clean segment within each second of the video and modelling the relation with time for bins with at least 5 segments of clean data, fitted with a linear and a quadratic model.

**Fig. 2:**
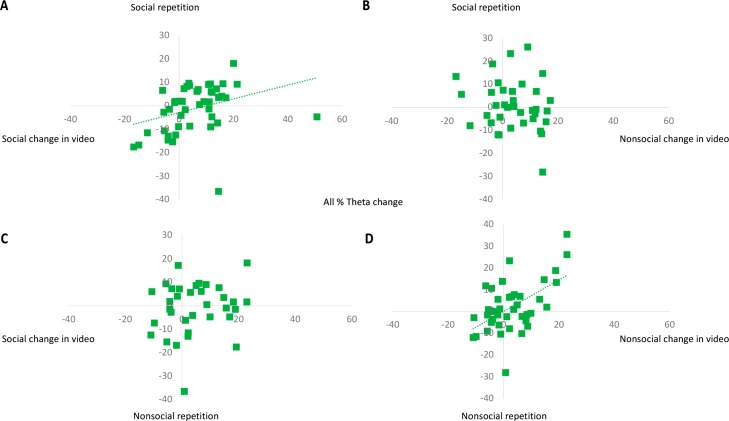
Violin plot showing the distribution and mean of Pearson *r-*values for individual correlations between frontal theta power and segment number.

As expected, Index B allowed us to include more infants (n = 47, 50.5 % of the full sample who came for the visit at 6-months, 48.5 % of the full sample from whom some EEG was gathered). We first established that the linear assumptions underpinning Index B were met by testing the fit of a linear vs a quadratic polynomial on data from the collapsed sample (generated by averaging data from each infant who provided a clean segment within each second of the video and modelling the relation with time for bins with at least 5 segments of clean data). The linear model fit well (*F*(1,113) = 57.37, *p* < 0.001; adj r^2^ = 0.333; standardised beta = 0.58; *t*(113) = 7.57, *p* < 0.001). The quadratic model explained less variance (adj r^2^ = 0.327; *F*(1,113) = 28.51, *p* < 0.001), and the quadratic component was not significant (standardised beta = -0.18; *t*(113) = -0.33, *p* = 0.75). The use of a linear model was thus supported. Fig. 1 displays these models. Pearson correlations were then computed between segment number and theta power for each individual participant. In the following analyses, using Fisher-transformed data, as is recommended for smaller sample sizes (Silver and Dunlap, 1987), provided nearly identical results (see, SM 2.1). A one-sample *t*-test was performed on the resulting individual *r*-values, which revealed that *r-*values were significantly different from zero *t*(46) = 4.34, *p*  < 0.001, *d* = 0.63, 95 % CI [0.06, 0.15]; Fig. 2. The mean *r-*value was positive (*M* = 0.10, *SD* = 0.16), indicating that frontal theta power increased over the course of the video in infants as a group. Since Index B was sensitive to the phenomenon of interest and allowed us to include a greater proportion of infants than Index A, we used Index B for the correlations with cognitive performance measures. Choosing Index B conformed to the procedure outlined in the pre-registration. Using Spearman correlation for Index B (instead of Pearson correlation) yielded nearly identical results to the ones reported in the following (see, SM 2.2).

### 3.2 Frontal theta power as a predictor of cognitive and executive function skills

**Fig. 3:**
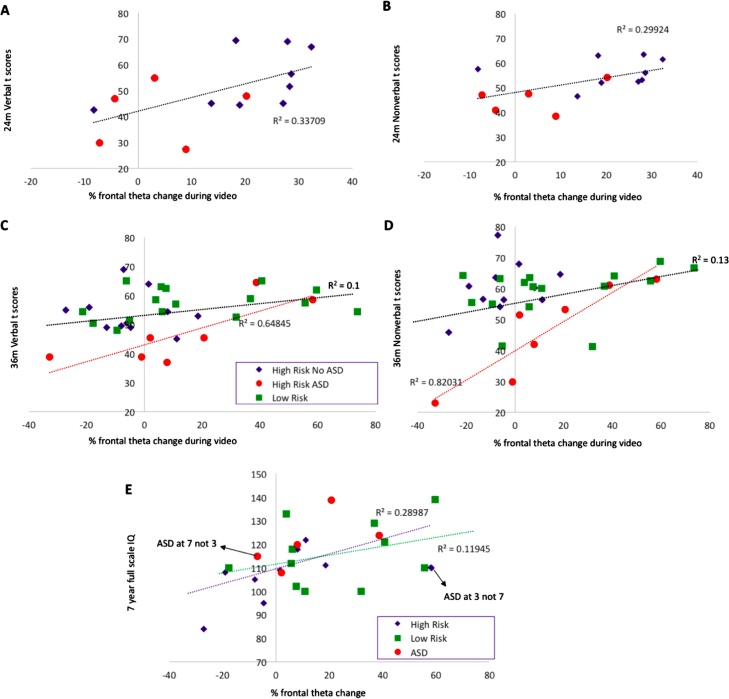
Association between frontal theta change at 6 months and (A) MSEL Non-verbal skills at 9 months and (B) MSEL Visual Reception skills at 9 months.

The magnitude of association between frontal theta power and time (how long the infant had been presented with the video for) was positively correlated with non-verbal cognitive level at 9 months *r*(40) = 0.302, *n* = 42, *p* = 0.026, 95 % CI [-0.002, 0.555], Fig. 3A; indicating that, as predicted, a stronger association between frontal theta power and video time was predictive of higher non-verbal cognitive ability. Frontal theta power was not significantly associated with inhibitory control as assessed by the Freeze-Frame task at 6 and 9 months, *r*(36) = 0.09, *n* = 38, *p* = 0.289, 95 % CI [-0.23, 0.40] and *r*(38) = 0.09, *n* = 40, *p* = 0.288, 95 % CI [-0.23, 0.39], or with performance on the A-not-B task at 9 months, *r*(38) = -0.158, *n* = 40, *p* = 0.165, 95 % CI [-0.45, 0.16]. See SM 2.3 for frontal theta change results for subsets of participants included in each analysis. See SM 2.4–2.10 for other additional analyses.

### 3.3 Exploratory analyses

To further investigate the link between frontal theta power change and cognitive ability, we separately examined the sub-components of the MSEL non-verbal cognitive scale: Fine Motor and Visual Reception. A two-tailed Pearson correlation revealed that frontal theta power change at 6 months significantly predicted Visual Reception abilities, *r*(40) = 0.50, *n* = 42, *p* = 0.001, 95 % CI [0.24, 0.70], Fig. 3B, but not Fine Motor abilities, *r*(40) = 0.01, *n* = 42, *p* = 0.936, 95 % CI [-0.29, 0.32], at 9 months.

## 4 Discussion

Though frontal theta power has previously been linked to learning and memory (Hsieh and Ranganath, 2014), little work has investigated the potential link between frontal theta and individual differences in cognitive function. In this pre-registered study, we first developed a new index of engagement with a novel stimulus (a dynamic non-social video) by examining the strength of association between theta power and video viewing time. The new index showed that theta power increases during the course of video presentation, consistent with previous work (Jones et al., 2020), and resulted in substantially less data attrition in young infants. We then demonstrated that individual differences in this index at 6 months predicted non-verbal cognitive ability at 9 months. In contrast, frontal theta power change was not associated with early measures of executive function (EF).

The finding that individual differences in frontal theta modulation at 6 months predicts non-verbal cognitive ability at 9 months is consistent with work by Jones et al. (2020), who found similar effects cross-sectionally in typical 1-year-old infants and longitudinally in a cohort of children at risk of developing ASD. Theta oscillations across frontal brain regions have been associated with situations involving infant learning (Berger et al., 2006; Orekhova et al., 1999), and may underlie this learning in uncertain contexts, such as during encounters with novel environments and objects (Orekhova et al., 2006). An increase in frontal theta power has also been associated with different aspects of memory processing in adults (Jensen and Tesche, 2002; Long et al., 2014) and infants (Begus et al., 2015). In conjunction with the results of the current study, these findings support the role of frontal theta in learning, memory and attention. The exact mechanism underlying the reported association will require further investigation. Nevertheless, we suggest that the dynamic modulations in frontal theta power may indicate the coordination of multiple systems during learning about naturalistic events. That is, frontal theta may reflect integrative neural processes across attentional, perceptual and memory domains, which, when interrupted or compromised, may lead to sub-optimal basic cognitive abilities (i.e., memory, attention, prediction processing difficulties) in infancy and, subsequently, poor learning and cognitive outcomes later in development.

More research is needed to fully establish that early frontal theta power change is a suitable biomarker of cognitive ability into the later childhood years. However, the current study does support that modulation of frontal theta power already at 6 months predicts later infant non-verbal cognitive ability, which *may* in turn relate to later cognitive ability. Current behavioural measures of cognitive development are often influenced by a number of confounding factors, including the child’s compliance, the testing environment, and *what* the child has already learnt, which is highly influenced by experience. Cognitive measures in infancy tend to be particularly problematic because infants have limited language, motor and attention skills (Hendry et al., 2016; Holmboe et al., 2008). Using a more direct measure of the activation in brain systems involved in learning, such as frontal theta modulation, could overcome some of these obstacles and may have a significant practical impact in terms of identifying children at risk of cognitive delay earlier than previously possible. In addition, developing interventions which involve recording theta power and presenting information during optimal periods may enable early difficulties with theta processing to be somewhat targeted and countered before broader cognitive deficits emerge. Such work could also be helpful for measuring the effects of risk factors on brain development in resource-poor settings, where behavioural measures are more difficult to apply (Lloyd-Fox et al., 2014b).

The present study is the first to find that frontal theta modulation from as early as 6 months of age can predict later non-verbal abilities. Interestingly, 6 months is also the earliest point at which simple EF abilities, such as inhibitory control, have been shown to emerge (Holmboe et al., 2018a). Nevertheless, despite work indicating that frontal theta power is associated with cognitive control in adults (Cavanagh and Frank, 2014), pre-school children (Meyer, et al., 2019) and toddlers (Conejero et al., 2016), and the fact that the frontal cortex has often been associated with early EF (for review, see Diamond, 2002; Holmboe, 2017; Fiske and Holmboe, 2019), in the current study there was no indication that frontal theta modulation was associated with EF. It should, however, be noted that executive functions are still relatively immature in infancy, with substantial spurts in development seen across the pre-school years (Garon et al., 2008; Petersen et al., 2016). Furthermore, at present, it is unknown how the simple forms of EF that can be measured in infancy relate to later more complex EF (for discussion, see Holmboe et al., 2018a). It is therefore possible that associations between theta modulation in infancy and EF emerge at a later stage in development as these functions mature.

Alternatively, the finding that modulation of theta across the frontal scalp area is related to non-verbal cognitive ability rather than to early manifestations of working memory and inhibition could indicate that other brain areas play a key role in the observed effect. There is evidence, for example, that frontal theta may be driven by hippocampal theta (Lega et al., 2012). Given that the hippocampus is implicated in information consolidation and memory, frontal theta may be part of a hippocampus-driven processing system linked specifically to learning mechanisms, and not to attentional or cognitive control per se, at least in infancy. Furthermore, Xie et al. (2018) found that the neural generators of theta power during sustained attention in infancy were different (orbito-frontal and temporal cortical areas) from those observed during adult tasks involving cognitive control (cingulate cortex), suggesting potentially different cognitive mechanisms associated with theta band activity at different ages.

Exploratory analyses of the two scales comprising the non-verbal MSEL score, indicated that the effect was specific to infants’ Visual Reception abilities. The correlation between frontal theta change at 6 months and Visual Reception at 9 months was *r* = 0.50 (*p* < 0.001), a moderate-to-large effect, whereas the equivalent correlation with the Fine Motor scale indicated no association, *r* = 0.01 (*p* = 0.94). These findings were exploratory and will therefore need replication in an independent sample. It is interesting to note, however, that the Visual Reception scale measures skills in visual discrimination, memory, organisation and processing, and thus may reflect infants’ abilities to process and consolidate visual information. Since the Visual Reception scale taps primarily non-verbal aspects of cognitive development, such as visuo-spatial abilities and early problem-solving skills, this finding could indicate that frontal theta modulation relates particularly closely to these aspects of development, which may later translate into fluid aspects of intelligence. The relationship between these skills and underlying neural processes such as frontal theta could be a fruitful area for future investigation.

Follow-up analyses indicated that frontal theta change predicted later non-verbal skills over and above both overt visual behaviour and average theta power during video viewing (SM 2.4, 2.5, 2.9). Infants’ amount of looking to the screen was not related to frontal theta power change and did not significantly predict later non-verbal skills. This is consistent with previous infant research suggesting that theta power change can index the level of cognitive processing independently of visual and manual exploration (Begus et al., 2011). Furthermore, the association between frontal theta power change and later non-verbal skills remained significant when controlling for average theta across the video presentation. These findings suggest that frontal theta power modulation is uniquely predictive of non-verbal cognitive ability and therefore a suitable candidate neural marker of early cognitive development.

A strength of the current study is that it was pre-registered on the OSF website, with a clear analysis plan that was followed meticulously to maintain openness and transparency (Holmboe et al., 2018b). Furthermore, the study benefitted from using well-established measures of cognitive development and early EF (Mullen, 1995; Holmboe et al., 2018a), and from a longitudinal design that precluded effects being specific to circumstances (e.g., mood) on the day of EEG testing. The study also has a number of limitations. Firstly, though the longitudinal design does suggest a possible causal link between early frontal theta power change and subsequent cognitive ability, further longer-term longitudinal work involving both EEG and cognitive assessments at each time point are needed to confirm this. Secondly, only a single video stimulus (a dynamic non-social video) was used to investigate frontal theta change in the current study. Although previous studies have clearly indicated that frontal theta increase can be observed across a range of stimuli and processing demands (Begus et al., 2011; Meyer et al., 2019; Stroganova & Orekhova, 2007), it will be important to establish in future research the exact time course and other key parameters for this effect. For example, what is the optimal stimulus to detect this effect, when during stimulus processing does the theta increase ‘peak’, and what is the best theta-change time window for obtaining a neural marker of later cognitive development? Work which makes use of Neuroadaptive Bayesian optimisation (that is, using machine learning techniques to adapt a stimulus in real-time until an optimal brain response is found (Lorenz et al., 2017)) may be used to identify the stimuli which elicit the greatest theta change and could be helpful in answering these questions. Thirdly, the sample of the current study was relatively small (although reasonable for an infant EEG study) and largely consisted of infants from high socio-economic status families (see Table 1), who may not be representative of the wider population. Finally, even using a new, improved index of theta change in this study, attrition rate remained relatively high (around 50 %). This is particularly important when considering how frontal theta may be used as a diagnostic tool or a target for intervention. Methods which improve data capture, such as carrying out multiple sessions and using better EEG measurement systems, ought to be trialled in order to improve this and to increase the feasibility of using theta change for identification and intervention.

Whilst the focus of the current study was on frontal theta power, it is likely that other components of infant EEG correlate with important aspects of development. An extensive literature exists linking alpha power to early EF (e.g., Bell and Fox, 1992; Wolfe and Bell, 2007; Cuevas et al., 2012), and a recent study by Perone et al. (2018) found a clear association between theta/beta ratio and EF across early and middle childhood. Future studies should investigate multiple mechanistically relevant brain biomarkers in relation to key cognitive outcomes.

Nonetheless, the findings of the present study confirm the feasibility of identifying neural correlates that predict later cognitive ability from early infancy, an important first step for the potential identification of and intervention for delayed cognitive development.

## Data statement

The data that support the findings of this study are openly available in anonymised form on OSF: https://osf.io/8yhwe/.

## CRediT authorship contribution statement

**Eleanor K. Braithwaite:** Conceptualization, Formal analysis, Methodology, Visualization, Writing - original draft, Writing - review & editing. **Emily J.H. Jones:** Conceptualization, Formal analysis, Methodology, Supervision, Visualization, Writing - review & editing. **Mark H. Johnson:** Conceptualization, Funding acquisition, Methodology, Supervision, Writing - review & editing. **Karla Holmboe:** Conceptualization, Data curation, Formal analysis, Funding acquisition, Investigation, Methodology, Project administration, Supervision, Writing - review & editing.

## Declaration of Competing Interest

The authors whose names are listed above certify that they have no affiliations with or involvement in any organisation or entity with any financial interest (such as honoraria; educational grants; participation in speakers’ bureaus; membership, employment, consultancies, stock ownership, or other equity interest; and expert testimony or patent-licensing arrangements), or non-financial interest (such as personal or professional relationships, affiliations, knowledge or beliefs), in the subject matter or materials discussed in this manuscript.

The authors declare that they have no known competing financial interests or personal relationships that could have appeared to influence the work reported in this paper.

## Footnotes

Appendix A Supplementary material related to this article can be found, in the online version, at doi: https://doi.org/10.1016/j.dcn.2020.100818.
