# Supplementary material for: Dynamic modulation of frontal theta power predicts cognitive ability in infancy
Source: Dev Cogn Neurosci. 2020 Jul 8;45:100818. doi: 10.1016/j.dcn.2020.100818 (PMC7393453; doi:10.1016/j.dcn.2020.100818)
Supplement: Supplementary file 1 [file mmc1.docx]

Supplementary Materials

1. **Additional Methods**

1.1 EEG

*1.1.1 Screenshots from the non-social video which participants watched during EEG recording.*

*
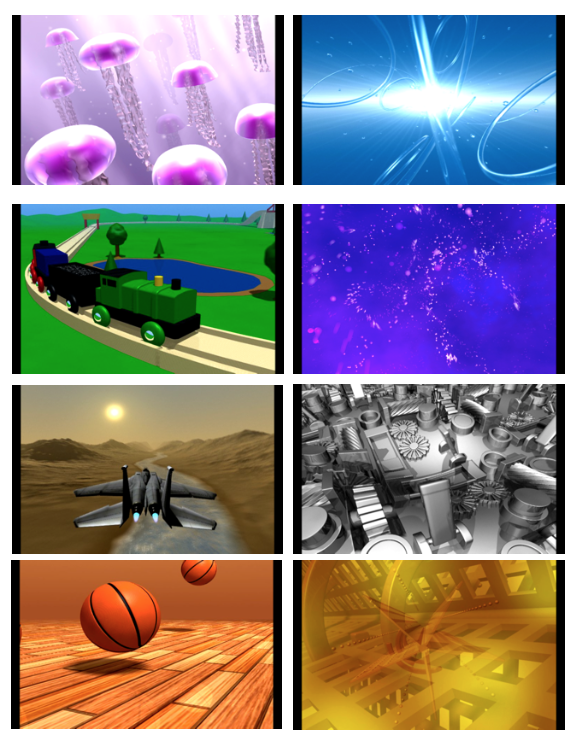
*

*1.1.2 A copy of the non-social video which participants watched during EEG recording*

The video can be accessed here: <https://osf.io/829tz/>

*1.1.3 Topographical area:* The topographical area used in the present study was a fronto-central region consisting of the following channels: 11, 12, 13, 9, 7, 8, 3, 4, 6, 2, 62, 58 (Figure S1).

*Figure S1:* Scalp map for 64-channel EGI sensor net displaying the channels used


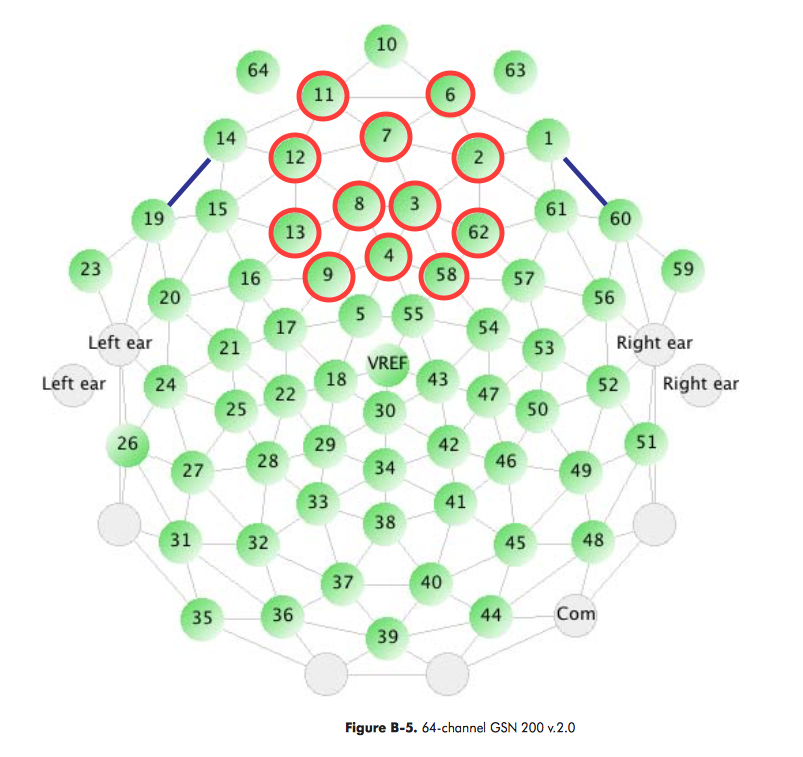


*1.1.4 Methods for artefact detection*: EEG data was segmented into 1-s segments. Artefacts were identified using both automatic detection software (NetStation 4.5.7) and through hand-editing (E. Braithwaite). Segments were rejected if the signal amplitude exceeded 250µV, if electro-ocular, movement or muscular artefact occurred, or if 22% of channels (or more than five within a scalp region) were subject to interpolation. Bad channels for the remaining segments were then interpolated by an algorithm in Netstation 4.5.7 and data was re-referenced to the average, before being imported into Matlab.

*1.1.5 EEG exclusion criteria:* As video viewing was the first task at 6 months, it is likely that there was initially a significant amount of movement artefact caused by the caregiver settling into a comfortable position with their baby. It was therefore decided to use the first good segment of EEG recording as the starting point for the analyses, as an indicator of the point at which infants were settled and able to watch the video (i.e., the start of calm video viewing). The ‘first good’/‘first clean’ segment was the first second-long segment of EEG data that was not rejected according to the rejection criteria (see section 1.1.4). Infants were excluded if the first good segment occurred later than the 45^th^ second (6 infants were excluded according to this criterion), as they were deemed not to have seen enough of the video to induce theta change. The first clean EEG segment was used as the starting point for both Indices A and B.

1. **Additional Results**

2.1 Fisher transformation results: For Index B, Fisher’s transformation was also performed to normalise the individual Pearson *r*-values for the correlation between segment number and theta power, as is recommended for smaller sample sizes (Silver & Dunlap, 1987). The following analyses were then carried out (mirroring the analyses of non-transformed data reported in the paper):

*2.1.1 Change in frontal theta power*: A one-sample t-test was performed, which revealed that Fisher-transformed *r-*values were significantly different from zero *t*(46) = 4.30, *p* < .001, *d* = 0.63, 95% CI [0.06, 0.16]. The mean Fisher-transformed *r-*value for the group was larger than zero (*M* = 0.11, *SD* = 0.17), indicating that frontal theta power increased over the course of the video.

*2.1.2 Change in frontal theta power predicting cognitive ability*: A positive correlation between Fisher-transformed *r*-values and non-verbal cognitive level was also found to be significant, *r*(40) = .31, *n* = 42, *p* = .024 (one-tailed), 95% CI [0.003, 0.56], indicating that, as predicted, higher frontal theta power change was associated with higher non-verbal cognitive ability.

*2.1.3 Change in frontal theta power predicting executive function:* Fisher-transformed *r*-values were not associated with inhibitory control as assessed by the Freeze-Frame task at 6 or 9 months, *r*(36) = .10, *n* = 38, *p* = .285 (one-tailed), 95% CI [-0.23, 0.40] and *r*(38) = .09, *n* = 40, *p* = .301 (one-tailed), 95% CI [-0.23, 0.39], respectively, or with A-not-B performance, *r*(38) = -.15, *n* = 40, *p* = .174 (one-tailed), 95% CI [-0.44, 0.17].

*2.1.4 Exploratory analyses:* A final set of correlations were carried out exploring the association between Fisher-transformed *r*-values and the two separate components of the MSEL non-verbal cognitive scale: Fine Motor and Visual Reception. A two-tailed Pearson correlation revealed that frontal theta power change at 6 months significantly predicted Visual Reception abilities, *r*(40) = .50, *n* = 42, *p* = .001, 95% CI [0.24, 0.70], but not Fine Motor abilities, *r*(40) = .02, *n* = 42, *p* = .90, 95% CI [-0.29, 0.32], at 9 months.

2.2 Spearman correlation analysis: Spearman correlation was also carried out between segment number and theta power for each individual participant to confirm that the results were unchanged when theta change was indexed by a non-parametric test:

*2.2.1 Change in frontal theta power:* A one-sample t-test was performed, which revealed that Spearman *r-*values were significantly different from zero *t*(46) = 3.69, *p* = .001, *d* = 0.54, 95% CI [0.04, 0.15]. The mean *r-*value for the group was larger than zero (*M* = 0.10, *SD* = 0.18), confirming that frontal theta power increased over the course of the video.

*2.2.2 Change in frontal theta power predicting cognitive ability:* A one-tailed Pearson correlation indicated a positive association between Spearman *r*-values for frontal theta change at 6 months and non-verbal cognitive ability at 9 months, *r*(40) = .30, *n* = 42, *p* = .027, 95% CI [-0.005, 0.55]. This confirmed that increasing frontal theta power was associated with higher non-verbal cognitive ability, in line with the equivalent analyses carried out with the Pearson-based *r*-values between segment number and frontal theta power.

*2.2.3 Change in frontal theta power predicting executive function:* Spearman *r*-values were not associated with inhibitory control as assessed by the Freeze-Frame task at 6 or 9 months, *r*(36) = .214, *n* = 38, *p* = .098 (one-tailed), 95% CI [-0.12, 0.52] and *r*(38) = .23, *n* = 40, *p* = .077 (one-tailed), 95% CI [-0.05, 0.45], respectively, or with A-not-B performance, *r*(38) = -.09, *n* = 40, *p* = .298 (one-tailed), 95% CI [-0.43, 0.29].

*2.2.4 Exploratory analyses:* A final set of correlations were carried out exploring the association between Spearman *r*-values and the two separate components of the MSEL non-verbal cognitive scale: Fine Motor and Visual Reception. A two-tailed Pearson correlation revealed that frontal theta power change at 6 months significantly predicted Visual Reception abilities, *r*(40) = .53, *n* = 42, *p* < .001, 95% CI [0.35, 0.71], but not Fine Motor abilities, *r*(40) = -.02, *n* = 42, *p* = .927, 95% CI [-0.33, 0.30], at 9 months.

2.3 Frontal theta change for subsets of participants included in each analysis: Some participants did not have data for frontal theta power change and each of the behavioural tasks, therefore the analysis of the association between frontal theta power and segment number was repeated for these four subsets of participants. Table S1 shows the average number of EEG trials (1-s segments) included in the Index B analysis for participants in the full cohort and in each of the subsets. For each of these subsets, Index B was recalculated and one-sample t-tests confirmed that frontal theta power significantly increased in each. Table S2 shows the results of the t-tests.

Table S1: *Means, standard deviations and ranges of the number of clean and usable EEG trials for the full Index B cohort and four subsets of participants who had data for frontal theta power change and each of the behavioural tasks. Each subset includes the participants who had data available from that behavioural task, i.e., the ‘Non-verbal subset’ includes participants who had a non-verbal MSEL score.*

| Group | *N* | *M* | *SD* | Range  Minimum Maximum |
| --- | --- | --- | --- | --- |
| Full Index B cohort | 47 | 38.06 | 22.58 | 10 106 |
| Non-verbal subset | 42 | 37.24 | 22.98 | 10 106 |
| A-not-B subset | 40 | 36.15 | 22.97 | 10 106 |
| Freeze-Frame at 6m/o subset | 38 | 38.71 | 22.90 | 10 106 |
| Freeze-Frame at 9m/o subset | 40 | 37.45 | 23.54 | 10 106 |

Table S2: *t-value, degrees of freedom, p-value, mean, standard deviation, Cohen’s d and 95% confidence intervals for one-sample t-tests performed on Pearson r-values of frontal theta change* *for each of the subsets of participants with data for frontal theta power change and each of the behavioural tasks. Each subset includes the participants who had data available from that behavioural task, i.e., the ‘Non-verbal subset’ includes participants who had a non-verbal MSEL score.*

| Subset | *t* | *df* | *p* | *M* | *SD* | *d* | 95% CI  Minimum Maximum |
| --- | --- | --- | --- | --- | --- | --- | --- |
| Non-verbal subset | 3.93 | 41 | <.001 | 0.11 | 0.17 | 0.61 | 0.05 0.15 |
| A-not-B subset | 3.78 | 39 | .001 | 0.10 | 0.17 | 0.60 | 0.05 0.16 |
| Freeze-Frame at 6m/o subset | 3.28 | 37 | .002 | 0.08 | 0.16 | 0.53 | 0.03 0.14 |
| Freeze-Frame at 9m/o subset | 3.82 | 39 | <.001 | 0.10 | 0.17 | 0.60 | 0.05 0.16 |

2.4 Looking time analyses. In order to address whether infants’ attentiveness to the video predicted frontal theta power change, non-verbal cognitive skill and/or executive functioning, additional analyses were performed with percentage looking time to the video. Video recordings of participants watching the stimulus video were coded offline using *Datavyu.* Videos were aligned with the EEG analyses such that only data from the first good segment of EEG to the end of the video were included, as in index B. Over the course of video presentation, videos were coded according to whether the infant’s eye-gaze was upon the screen or elsewhere. In three of the video recordings, the stimulus video playback was briefly stopped and started again due to a minor technical problem; these participants have not been included in the looking time analyses in order to ensure that all video recordings were on the same timescale as the theta change measure for these analyses. Videos were segmented into video frames, which were each 40 milliseconds long. Each video frame was coded according to whether infants were looking at the screen or not. Percentage looking time was then calculated in terms of how many milliseconds the infant spent looking at the screen versus elsewhere during the presentation of the stimulus video. Analyses of the associations between percentage looking time and each of frontal theta power change, non-verbal cognitive skill, executive functioning and Visual Reception ability were then conducted.

*2.4.1 Percentage looking time related to frontal theta power change.* For comparability with the pre-registered analyses and the analyses in 2.4.2-2.4.5 (below), this analysis was run for the subset of participants who had complete theta change and non-verbal skills data. A two-tailed Pearson correlation between percentage looking to the stimulus video and frontal theta power change was not significant at the *p* < .05 significance level, *r*(37) = .22, *n* = 39, *p* = .174, 95% CI [-0.096, 0.49].

*2.4.2 Percentage looking time related to non-verbal cognitive ability.* A two-tailed Pearson correlation between percentage looking time and non-verbal cognitive ability was not significant at the *p* < .05 significance level, *r*(37) = -.05, *n* = 49, *p* = .758, 95% CI [-0.31, 0.24].

*2.4.3 Percentage looking time related to executive functioning.* Two-tailed Pearson correlations between percentage looking time and inhibitory control as assessed by the Freeze-Frame task at 6 and 9 months, *r*(33) = -.14, *n* = 35, *p* = .420, 95% CI [-0.49, 0.20] and *r*(35) = .21, *n* = 37, *p* = .212, 95% CI [-0.09, 0.46], and the A-not-B task at 9 months, *r*(35) = .23, *n* = 37, *p* = .172, 95% CI [-0.06, 0.47], were not significant at the *p* < .05 significance level.

*2.4.4 Percentage looking time related to Visual Reception abilities.* A two-tailed Pearson correlation between percentage looking time and Visual Reception abilities at 9 months was not significant, *r*(37) = -.02, *n* = 39, *p* = .918, 95% CI [-0.28, 0.29].

Taken together, these analyses suggest that frontal theta power change captures an effect, i.e., the significant association between frontal theta power change at 6 months and non-verbal cognitive ability at 9 months, which measures of looking behaviour do not.

2.5 Looking time change analyses. In order to address whether change in infants’ attentiveness to the video predicted frontal theta power change, non-verbal cognitive skill and/or executive functioning, additional analyses were performed with change in looking time to the video. Video recordings of participants watching the stimulus video were coded offline using *Datavyu.* Videos were aligned with the EEG analyses such that only data from the first good segment of EEG to the end of the video were included, as with the EEG included in index B. Three video recordings were not included due to a brief interruption in the video playback (see section 2.4). Included videos were segmented into video frames, which were each 40 milliseconds long. Each video frame was coded according to whether infants were looking at the screen or not. Every 1s segment of video was then coded as ‘looking’ or ‘not looking’ based upon the percentage of frames in which participants were looking or not. If participants were looking at the screen for over 50% of the video frames in a 1s segment, that 1s segment of video was coded as ‘looking’, whereas less than 50% of frames with gaze on the screen in a 1s segment was coded as ‘not looking’. A Pearson correlation was conducted between segment number (i.e., each second of the video) and these values. Pearson r-values were used as a measure of change in looking time over the course video. Analyses of the associations between change in looking time and each of frontal theta power change, non-verbal cognitive skill and executive functioning were then conducted.

*2.5.1 Change in looking time.* A one sample t-test comparing *r*-values of looking time change to zero was significant, *t*(38) = -2.75, *p* = .009 (two-tailed), *d* = -0.44, 95% CI [-0.18, -0.03], with the mean indicating that looking decreased over the course of video viewing, *M* = -0.11, *SD* = 0.24.

*2.5.2 Looking time change related to frontal theta power change.* For comparability with the pre-registered analyses and the analyses in 2.5.3-2.5.5 (below), this analysis was run for the subset of participants who had complete theta change and non-verbal skills data*.* A two-tailed Pearson correlation between change in looking time to the stimulus video and frontal theta power change was not significant at the *p* < .05 significance level, *r*(37) = -.08, *n* = 39, *p* = .628, 95% CI [-0.44, 0.30].

*2.5.3 Looking time change related to non-verbal cognitive ability.* A two-tailed Pearson correlation between looking time change and non-verbal cognitive ability was not significant at the *p* < .05 significance level, *r*(37) = .16, *n* = 39, *p* = .342, 95% CI [-0.23, 0.52].

*2.5.4 Looking time change related to executive functioning.* Two-tailed Pearson correlations between looking time change and inhibitory control as assessed by the Freeze-Frame task at 6 and 9 months, *r*(33) = .03, *n* = 35, *p* = .872, 95% CI [-0.29, 0.33] and *r*(35) = .04, *n* = 37, *p* = .808, 95% CI [-0.28, 0.35], and the A-not-B task at 9 months, *r*(35) = .17, *n* = 37, *p* = .303, 95% CI [-0.08, 0.42], were not significant at the *p* < .05 significance level.

*2.5.5 Looking time change related to Visual Reception abilities.* A two-tailed Pearson correlation between change in looking time and Visual Reception skills was not significant, *r*(37) = -.01, *n* = 39, *p* = .937, 95% CI [-0.30, 0.30].

These analyses rule out the possibility that change in infants’ looking behaviour was the driving factor in the association between frontal theta power change at 6 months and non-verbal cognitive ability at 9 months. Only frontal theta power change was predictive of later non-verbal skills.

2.6 Percentage of good EEG segments analyses: In order to address whether there was an association between artefact-free trials and frontal theta power change, non-verbal cognitive skills and/or executive functioning, additional analyses were performed. Furthermore, we were interested in whether there was a significant positive association between the proportion of artefact-free trials and looking time, as this would suggest that EEG data quality can be used as a proxy measure for looking time. We calculated the percentage of good segments as a proportion of total EEG segments per infant; associations with percentage looking time, frontal theta power change, non-verbal cognitive skills and executive functioning were then assessed.

*2.6.1 Percentage of good EEG segments related to frontal theta power change.* For comparability with the pre-registered analyses and the analyses in 2.6.2-2.6.4 (below), this analysis was run for the subset of participants who had complete theta change and non-verbal skills data. A two-tailed Pearson correlation between percentage of good EEG segments and frontal theta power change was not significant at the *p* < .05 significance level, *r*(40) = .16, *n* = 42, *p* = .317, 95% CI [-0.14, 0.42].

*2.6.2 Percentage of good EEG segments related to non-verbal cognitive ability.* A two-tailed Pearson correlation between percentage of good EEG segments and non-verbal cognitive ability was not significant at the *p* < .05 significance level, *r*(40) = .027, *n* = 42, *p* = .867, 95% CI [-0.27, 0.28].

*2.6.3 Percentage of good EEG segments related to executive functioning.* A two-tailed Pearson correlation between percentage of good EEG segments and inhibitory control as assessed by the Freeze-Frame task at 6 month was significant, *r*(36) = -.35, *n* = 38, *p* = .03, 95% CI [-0.61, -0.08] at the *p* < .05 significance level. Two-tailed Pearson correlations between percentage of good EEG segments and Freeze-Frame performance at 9 months, *r*(38) = .118, *n* = 40, *p* = .470, 95% CI [-0.24, 0.47], as well as A-not-B performance at 9 months, *r*(38) = .018, *n* = 40, *p* = .914, 95% CI [-0.21, 0.46], were not significant.

*2.6.4 Percentage of good EEG segments related to Visual Reception abilities.* A two-tailed Pearson correlation between percentage of good EEG segments and Visual Reception abilities was not significant, *r*(40) = -.01, *n* = 42, *p* = .945, 95% CI [-0.28, 0.25].

The only correlation which reached significance at the *p* < .05 significance level was that between percentage of good EEG segments and Freeze-Frame performance at 6 months, however no relation was found between frontal theta power change and Freeze-Frame performance at 6 months, therefore this does not appear to be a confounding factor. Since the Freeze-Frame task was presented immediately after participants watched the stimulus video, on-the-day factors, such as alertness, mood and interest in the screen, may explain this association, especially as there is no significant association between percentage of good EEG segments (at 6 months) and Freeze-Frame performance at 9 months.

2.7 Percentage of good EEG segments related to percentage looking time. For comparability with the pre-registered analyses and the analyses in 2.6.2-2.6.4 (above), this analysis was run for the subset of participants who had complete theta change and non-verbal skills data. A two-tailed Pearson correlation between the percentage of good EEG segments and percentage looking time was highly significant, *r*(38) = .56, *n* = 40, *p* < .001, 95% CI [0.37, 0.72].

This suggests that good EEG segments may be an effective proxy for attentiveness to the screen. Since movement artefacts are a significant cause of infant EEG data loss, good segments of EEG may represent epochs in which infants were looking to the screen and were calm and attentive (similar to focussed attention in Lawson & Ruff, 2004; Jones et al., 2020).

2.8 Variance in theta power: Analyses were conducted in order to establish whether variance in theta power might be associated with theta power change or the percentage of clean and usable segments of EEG. These analyses were run for the subset of participants who had complete theta change and non-verbal skills data.

*2.8.1 Variance in theta power in relation to theta power change*. A two-tailed Pearson correlation between the amount of variance (standard deviation) in theta power and frontal theta power change was found to be non-significant, *r*(40) = .06, *n* = 42, *p* = .719, 95% CI [-0.27, 0.40]. This supports the conclusion that Index B measures a change in theta power which occurs over the course of video viewing, rather than random fluctuations in frontal theta power.

*2.8.2 Variance in theta power in relation to percentage good EEG segments.* A two-tailed Pearson correlation between the amount of variance (standard deviation) in theta power and percentage good EEG provided by each infant did not reach significance at the *p <* .05 significance level, *r*(40) = .09, *n* = 42, *p* = .587, 95% CI [-0.16, 0.35], indicating that the variability of theta power was not significantly related to the percentage of good segments that an individual infant provided. This suggests that it is not just that children who provided less usable EEG showed less (or more) variation in theta power and indicates that Index B measures change in theta power rather than variation in data quality within our sample.

2.9 Average theta power analysis: Additional analyses were carried out using a different measure of theta power (average theta power), which has been used in previous research (e.g., Jones et al., 2015; Dawson et al., 2012), for comparison with the proposed measure of frontal theta power change. These analyses were run for the subset of participants who had complete theta change and non-verbal skills data.

*2.9.1 Average theta power related to frontal theta power change.* A two-tailed Pearson correlation between average frontal theta power over the whole video from the first good segment of EEG and Pearson *r*-values of frontal theta change was not significant at the *p* < .05 significance level, *r*(40) = .20, *n* = 42, *p* = .206, 95% CI [-0.08, 0.47]. This suggests that frontal theta power change taps something different to traditional measures of theta power and adds value to the use of Index B in our analyses.

*2.9.2 Association between average theta power and non-verbal cognitive ability.* An exploratory two-tailed Pearson correlation between average theta power over the whole video and non-verbal cognitive level at 9 months was not significant at the *p* < .05 significance level, *r*(40) = .267, *n* = 42, *p* = .087, 95% CI [-0.040, 0.528]. However, there was a trend in the same direction as observed for frontal theta change (i.e., higher average theta tended to be associated with better non-verbal cognitive abilities), and if this measure had been proposed in the pre-registration instead of frontal theta change, this result would also have been significant (using a one-tailed test). We therefore conducted a partial correlation analysis to further address the specificity of frontal theta power change in predicting non-verbal cognitive ability. A partial Pearson correlation was conducted between Pearson *r*-values of frontal theta change and non-verbal score whilst controlling for average theta power. Since this analysis was in keeping with our original prediction about the relation between theta power change and non-verbal skill, and the direction of this association had been confirmed, a one-tailed test was performed. The correlation was found to be only slightly reduced and still significant at the *p* < .05 significance level, *r*(40) = .264, *n* = 42, *p* = .048, 95% CI [-.075, .525], suggesting that the relationship between frontal theta power change and non-verbal cognitive ability exists even when average theta power is held constant. This supports the conclusion that frontal theta power change captures something additional to average theta power. Additionally, a two-tailed partial Pearson correlation was conducted between average theta power over the whole video and non-verbal score whilst controlling for Pearson *r*-values of frontal theta change. This was not significant at the *p* < .05 significance level, *r*(39) = .221, *n* = 42, *p* = .164, 95% CI [-.079, .453], suggesting a lack of association between average frontal theta power and non-verbal ability when frontal theta power change is held constant, and further supporting the predictive advantage of frontal theta power change.

*2.9.3 Association between average theta power and Visual Reception ability.* A two-tailed partial Pearson correlation was conducted between Pearson *r*-values of frontal theta change and Visual Reception ability whilst controlling for average theta power over the whole video. The correlation was found to be only slightly reduced and still significant, *r*(39) = .486, *n* = 42, *p* = .001, 95% CI [-.075, .525], suggesting that the relationship between frontal theta power change and Visual Reception ability exists even when average theta power is held constant. Additionally, a two-tailed partial Pearson correlation was also conducted between average theta power over the whole video and Visual Reception abilities whilst controlling for Pearson *r*-values of frontal theta change. The correlation was not significant at the *p* < .05 significance level, *r*(39) = .083, *n* = 42, *p* = .607, 95% CI [-.346, .374]. This indicates that there is no significant association between average frontal theta power and Visual Reception ability when frontal theta power change is held constant. These results together support the conclusion that frontal theta power change captures something additional to average theta power and that it associates uniquely with early non-verbal cognitive skills.

2.10 Specificity Analyses: Additional analyses were carried out to explore the topographic specificity of our theta power change findings.

*2.10.1 Theta power change specificity analysis.* Pearson correlations were calculated between segment number and theta power for each electrode for each participant. Mean r-values were then calculated for each electrode. Figure S2 shows the topographic distribution of these mean r-values of theta power change. The yellow/orange colour over the majority of the scalp indicates a largely global increase in theta power during video viewing.

*2.10.2 Association between theta power change and non-verbal ability specificity analysis.* Correlations were also conducted between r-values of theta power change and non-verbal cognitive ability for each electrode: Figure S3 shows the topographic distribution of these mean r-values. The red colours over the frontal area indicates that the association between theta power change and non-verbal ability is strongest in frontal regions.

*Figure S2:* Topographical map of mean r-values of theta power change during video viewing. Red circles highlight the electrodes included in the fronto-central area of interest (as in Figure S1).


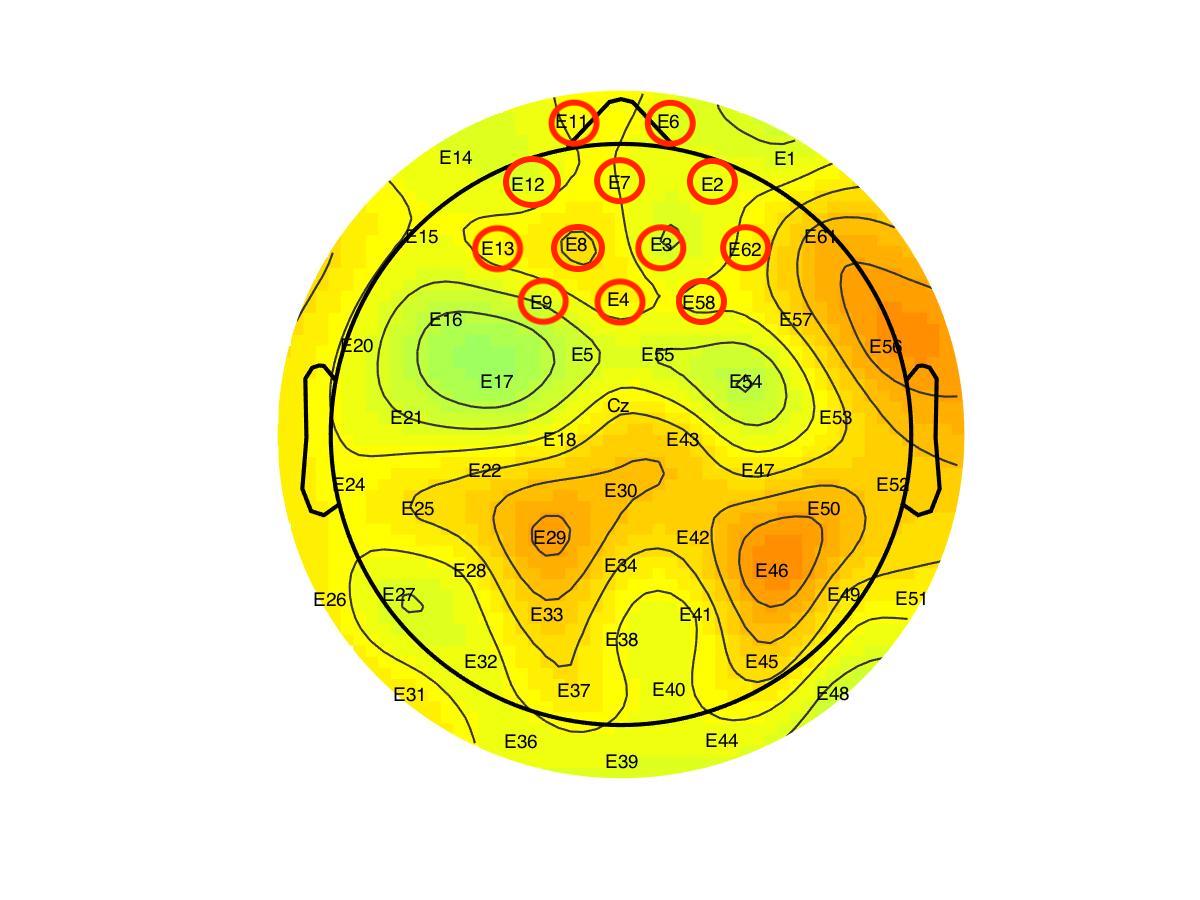


*Figure S3:* Topographical map of mean r-values for the association between theta power change and non-verbal ability. Pale pink circles highlight the electrodes included in the fronto-central area of interest (as in Figure S1).


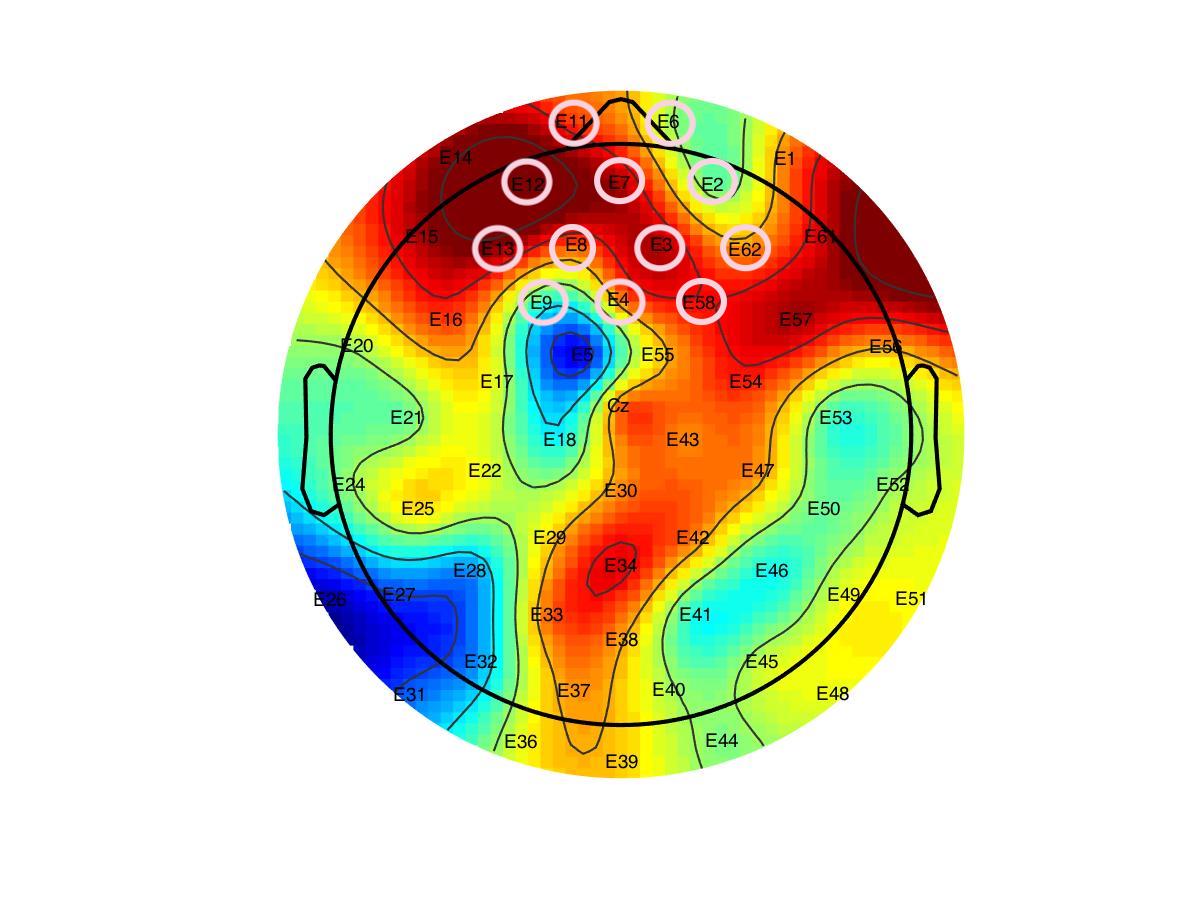


References

Dawson, G., Jones, E. J., Merkle, K., Venema, K., Lowy, R., Faja, S., ... & Smith, M. (2012). Early behavioural intervention is associated with normalized brain activity in young children with autism. *Journal of the American Academy of Child & Adolescent Psychiatry*, *51*(11), 1150-1159. <https://doi.org/10.1016/j.jaac.2012.08.018>

Jones, E. J. H., Goodwin, A., Orekhova, E., Charman, T., Dawson, G., Webb, S. J., & Johnson, M. H. (2020). Infant EEG theta modulation predicts childhood intelligence. *Scientific Reports*, *10*(1), 1-10. https://doi.org/10.1038/s41598-020-67687-y

Jones, E. J., Venema, K., Lowy, R., Earl, R. K., & Webb, S. J. (2015). Developmental changes in infant brain activity during naturalistic social experiences. *Developmental psychobiology*, *57*(7), 842-853. <https://doi.org/10.1002/dev.21336>.

Lawson, K. R., & Ruff, H. A. (2004). Early focused attention predicts outcome for children born prematurely. *Journal of Developmental & Behavioral Pediatrics*, *25*(6), 399-406. <https://doi.org/10.1097/00004703-200412000-00003>

Silver, N. C., & Dunlap, W. P. (1987). Averaging correlation coefficients: Should Fisher’s z transformation be used? *Journal of Applied Psychology*, *72*(1), 146–148. <https://doi.org/10.1037/0021-9010.72.1.146>
